# Supplementary material for: Metabolic Effects of Sodium Thiosulfate During Resuscitation from Trauma and Hemorrhage in Cigarette-Smoke-Exposed Cystathionine-γ-Lyase Knockout Mice
Source: Biomedicines. 2024 Nov 12;12(11):2581. doi: 10.3390/biomedicines12112581 (PMC11591741; doi:10.3390/biomedicines12112581)
Supplement: Supplementary file 1 [file biomedicines-12-02581-s001.zip › biomedicines-3260475-supplementary.pdf]

Supplements & Material

Analyzers and Devices	Vendor
ABL 800 Series Blood Gas Analyzer	Radiometer GmbH, Willich, Germany
Power Laboratoy 4/SP Converter	ADInstruments, Castle Hill, Australia
Flexivent Small Animal Ventilator	Scireq, MO, Canada
Disposable Pressure Transducer	FMI, Seeheim/ Oberbeerbach, Germany
MIO-501 DC Brückenmessverstärker	FMI, Seeheim/ Oberbeerbach, Germany
Syringe Pump 11	Havard Apparatus, Holliston, Massachusetts, USA
TKM-0903 Temperature Control	FMI, Seeheim/ Oberbeerbach, Germany
**Surgical Supplies**	
Arterial Catheter	FMI, Seeheim/ Oberbeerbach, Germany
Catheter for Vascular Access or Cannulation of Urinary Bladder (24GA 0.75IN)	Becton Dickinson Infusion Therapy Systems Inc., Sandy, UT, USA
Cannulas, Various Sizes	B Braun, Melsungen, Germany
Leukopor, Dressing	BSN medical GmbH, Hamburg, Germany
Syringes, Various Sizes	BD Plastik, Heidelberg, Germany
Sutures: 5.0 Prolene, 6.0 Ethilon, 8.0 Ethilon	Johnson & Johnson, Neuss, Germany
Polyester Membrane	Du Pont de Nemur, Bad Homburg, Germany
Tracheostomy Set (Custome Made): 18G Cannula and<br>Silicone Tubing	B Braun, Melsungen Germany<br>VWR International, Darmstadt, Germany

Central Venous Catheter (Custom Made): 30GA ½ Cannula and<br><br>Silicone Tubing	BD Microlance, Heidelberg, Germany<br><br>Bohlender, Grünsfeld, Germany
**Drugs**	
Buprenoprhine (Temgesic®)	Reckitt Bencksier, Slough, UK
Fentanyl (Fentanyl-Hameln®)	Hameln Pharma Plus GmbH, Hameln, Germany
Jonosteril 1/1 E	Fresenius Kabi, Bad Homburg, Germany
Ketamin (Ketanest-S®)	Pfizer, New York City, NY, USA
Midazolame (Midazolam-ratiopharm®)	Ratiopharm, Ulm, Deutschland
Norepinephrine (Arterenol®)	Sanovi-Aventis, Frankfurt am Main, Germany
Natriumhydroencarbonat 8,4%	D. Braun Melsungen AG, Melsungen, Germany
Sodium Heparine	D. Braun Melsungen AG, Melsungen, Germany
Oxygen	MTI Industriegase, Ulm, Germany
Sevoflurane	Abbott, Wiesbaden, Germany
Nitrogen	MTI Industriegase, Ulm, Germany
Tetraspan 6%	B. Braun, Melsungen, Germany
Sodium Thiosulfate 25%	Dr. Franz Köhler Chemie GmbH, Bensheim, Germany
**Mass Spectometry Supplies**	
13C6-Glucose, 6,6-2H2-Glucose<br><br>15N2-Urea	Campro Scientific, Berlin, Germany

2H5-Glycerole	
5,5,5-2H3-Leuzine	CIL, Tewksbury, MA, USA
2H3-Creatinine	CDN isotopes, Pointe-Claire, Quebec, Canada
Derivatizing Reagents	ABCR, Karlsruhe, Germany
Eppendorf-Cups	Eppendorf, Hamburg, Germany
Cation-exchange Extraction Cartridges	Phenomenex, Aschaffenburg, Germany
Falcon Tubes 15mL, 50mL	BD Biosciences, Bedford, USA
Pipette Tips	Eppendorf, Hamburg, Germany
6890/5973 GC/MS system	Agilent, Rialto, California, USA

**Chemical Supplies**	
10x TBS	24,2 g Tris<br>80g NaCl<br>pH balanced to pH =7.6<br>Final Volum1: 1l
10x PBS	95,5g PBS<br>Final Volume: 1l
